# Comparative Assessment of Commercial Collagen Peptides Following Simulated Gastrointestinal Digestion: Structural Stability, Bioactivity, and Digestibility

**DOI:** 10.3390/nu18142383

**Published:** 2026-07-21

**Authors:** Saeid Chobdar Rahim, Zehra Betül Ahi, Beyza Çay, Fatih Arıcan

**Affiliations:** 1 Collagen R&D Center, Collagen R&D Technology Inc., Istanbul 34959, Turkey; 2 Kazlıçesme R&D Center and Test Laboratory, Istanbul 34950, Turkey; zehraahi@kazlicesme.com.tr (Z.B.A.); beyzacay@kazlicesme.com.tr (B.Ç.); fatiharican@kazlicesme.com.tr (F.A.)

**Keywords:** collagen peptides, gastrointestinal digestion, degree of hydrolysis, antioxidant activity, fibroblast migration

## Abstract

**Background/Objectives**: Commercial collagen peptide products may differ in their structural characteristics and in vitro biological properties depending on manufacturing processes and hydrolysis conditions. However, comparative studies integrating simulated gastrointestinal digestion with structural, physicochemical, and cell-based analyses remain limited. This study comparatively evaluated commercially available bovine collagen peptide products marketed in Türkiye before and after simulated gastrointestinal digestion. **Methods**: Collagen peptide samples were characterized before and after simulated gastrointestinal digestion by determining hydroxyproline content, degree of hydrolysis (DH), molecular weight (MW) distribution, antioxidant activity, and fibroblast responses to investigate digestion-induced structural and biological changes. **Results**: Significant differences were observed among the evaluated products in hydroxyproline content, degree of hydrolysis, molecular weight distribution, antioxidant activity, and fibroblast responses. Simulated gastrointestinal digestion further hydrolyzed all collagen peptide samples, although the extent of structural modification varied among products. Antioxidant responses also differed following digestion, with some products maintaining relatively stable radical scavenging activity, whereas others exhibited increased or decreased antioxidant responses. Cell viability assays demonstrated that all collagen peptide samples were non-cytotoxic toward L929 fibroblasts under the experimental conditions. **Conclusions**: The findings indicate that simulated gastrointestinal digestion influences the structural characteristics and in vitro biological responses of commercial collagen peptide products to different extents. The observed differences among products suggest differential in vitro bioactivity associated with their structural characteristics and digestion behavior. Nevertheless, these findings are limited to in vitro observations and require confirmation through peptide characterization, bioavailability studies, animal models, and well-designed clinical investigations before conclusions regarding physiological efficacy or potential health benefits can be drawn.

## 1. Introduction

The skin acts as a dynamic biological barrier that continuously responds to both physiological and environmental stimuli throughout life. However, intrinsic aging, ultraviolet exposure, oxidative stress, and nutritional deficiencies progressively disrupt skin homeostasis, leading to structural and functional deterioration. One of the most prominent consequences of this process is the gradual decline in collagen content, which is essential for maintaining skin integrity, elasticity, and mechanical strength. As collagen loss accelerates with age, nutritional and biomedical strategies targeting collagen metabolism have attracted increasing scientific and industrial interest [1].

Collagen peptides are generated through the enzymatic hydrolysis of collagen obtained from connective tissue-rich sources, including bovine hide, porcine skin, and fish processing by-products. Owing to their reduced molecular weight, collagen peptides generally exhibit high water solubility and are readily digested and absorbed in the gastrointestinal tract. Their characteristic amino acid composition, particularly the high abundance of glycine, proline, and hydroxyproline residues, is closely associated with their biological functions related to skin elasticity, joint health, and bone metabolism. Beyond their structural role, collagen-derived peptides have attracted considerable attention due to their diverse bioactive properties, including antioxidant potential and other functional effects. Recent studies have suggested that collagen peptides may modulate cellular responses and exhibit various in vitro biological activities, highlighting their growing interest in functional food and nutraceutical research [2,3].

In addition to their nutritional value, gelatin-derived peptides have been widely reported to exhibit diverse biological activities, including antioxidant, antihypertensive, cholesterol-lowering, anticancer, and immunomodulatory effects. These bioactive peptide fractions, commonly referred to as biopeptides, consist of specific amino acid sequences capable of interacting with cellular and molecular targets, thereby modulating various physiological processes. Among their reported functions, antioxidant activity has received particular attention and is strongly influenced by peptide structure, molecular weight, and amino acid composition, especially the presence of hydrophobic and electron-donating residues that facilitate free radical scavenging. Furthermore, certain collagen-derived peptides have also demonstrated antimicrobial and antidiabetic potential, highlighting their multifunctional biological relevance. Among the available production strategies, enzymatic hydrolysis remains the most widely preferred approach due to its process efficiency, controllability, and ability to generate peptides with enhanced functional and bioactive properties [4,5,6,7,8].

Despite their widespread commercialization, collagen peptide products do not exhibit uniform structural or functional characteristics. Variations in raw material origin, enzymatic hydrolysis conditions, and downstream processing can substantially influence peptide composition, molecular weight distribution, gastrointestinal behavior, and biological activity. Among these parameters, molecular weight is considered particularly critical, as low-molecular-weight peptide fractions are generally associated with improved intestinal absorption and enhanced bioavailability [9,10]. However, extensive hydrolysis may also promote excessive peptide fragmentation, potentially disrupt functional amino acid sequences and reduce biological performance [11]. Consequently, the functional potential of collagen peptides appears to depend not only on peptide size, but also on the preservation of structurally active peptide fractions.

Although collagen peptides are increasingly incorporated into nutraceutical and functional food formulations, comparative studies directly evaluating commercially available products under simulated gastrointestinal conditions remain limited. Moreover, investigations integrating structural characterization, bioactivity, and cellular responses within a single experimental framework are still scarce. More importantly, the relationship between gastrointestinal digestion and the structure–function behavior of commercial collagen peptides remains insufficiently understood, particularly when assessed using combined physicochemical, bioactive, and cell-based approaches [2,12]. Therefore, comprehensive comparative studies are needed to better elucidate how hydrolysis profile and digestion stability influence the functional and biological performance of commercially available collagen peptide products.

Although the antioxidant and antimicrobial activities of collagen-derived peptides have been extensively reported, these functional properties alone do not fully represent their biological relevance under physiological conditions. A more comprehensive evaluation requires investigation at the cellular level to better understand peptide–cell interactions and their potential biological responses. In this context, cell-based assays such as the MTT assay provide valuable information regarding cellular metabolic activity and cell viability, thereby serving as an indirect indicator of cellular responses to peptide treatment [13,14]. Consequently, integrating structural, bioactive, and cell-based analyses may provide a more comprehensive understanding of the functional properties of collagen peptides.

The aim of this study was to comparatively evaluate commercially available collagen peptides in terms of their structural characteristics, bioactivity, and cellular responses before and after simulated gastrointestinal digestion. Differences in degree of hydrolysis, molecular weight distribution, amino acid composition, antioxidant activity, antimicrobial potential, and fibroblast behavior were investigated using an integrated analytical approach. In addition, cytocompatibility and wound-healing responses were evaluated using L929 fibroblast cells to further examine the biological relevance of peptide fractions following digestion. By combining physicochemical characterization with functional and cell-based analyses, this study provides a comprehensive assessment of the variability among commercial collagen peptide products and their potential implications for functional food and nutraceutical applications.

## 2. Materials and Methods

The collagen peptide samples evaluated in this study were obtained from commercially available products marketed in Türkiye through retail and pharmacy channels. According to the manufacturers’ specifications, all products were bovine hide-derived collagen peptides and did not contain added vitamins, minerals, flavoring agents, sweeteners, or other functional additives. Therefore, the samples were considered commercially available pure collagen peptide products. To ensure an objective comparative assessment, all products were anonymized and coded prior to analysis as RC, LC, HC, VC, and NC. All experimental procedures, data analysis, and result interpretation were performed using this coding system to minimize potential bias. Each commercial collagen peptide product evaluated in this study was represented by a single production batch. Therefore, potential batch-to-batch variability was not assessed and should be considered when interpreting the results.

### 2.1. Chemical Characterization

The proximate composition of collagen peptide samples was determined by analyzing crude protein, moisture, ash, lipid, and hydroxyproline contents according to standard procedures of the Association of Official Analytical Chemists (AOAC). Crude protein content was quantified using the Kjeldahl method based on total nitrogen determination with a nitrogen-to-protein conversion factor of 6.25. Moisture content was determined by oven drying at 105 °C until constant weight, whereas ash content was measured after complete mineralization at 550 °C using a muffle furnace. Lipid content was determined gravimetrically following petroleum extraction in a Soxhlet system.

Hydroxyproline, one of the characteristic amino acids of collagen, was quantified using a colorimetric method involving acid hydrolysis, oxidation, and subsequent reaction with Ehrlich’s reagent, with absorbance recorded at 558 nm. Collectively, these analyses provided a comprehensive physicochemical characterization of the commercially available collagen peptide samples.

### 2.2. In Vitro Gastrointestinal Digestion

In vitro gastrointestinal digestion of collagen peptide samples was performed according to the standardized INFOGEST 2.0 static digestion protocol described by Brodkorb et al. [15]. Initially, 1 g of collagen peptide powder was dissolved in 4 mL of distilled water and mixed with an equal volume of simulated salivary fluid (SSF) containing α-amylase to achieve a final enzymatic activity of 75 U/mL. The oral phase was carried out at pH 7.0 and 37 °C for 2 min under continuous agitation. For the gastric phase, the oral bolus was mixed (1:1, *v*/*v*) with simulated gastric fluid (SGF), and pepsin was added to obtain a final enzymatic activity of 2000 U/mL. The pH was adjusted to 3.0, and digestion was performed at 37 °C for 2 h under constant shaking. Subsequently, the gastric digest was mixed (1:1, *v*/*v*) with simulated intestinal fluid (SIF). Pancreatin was added to provide a final trypsin activity of 100 U/mL together with bile salts at a final concentration of 10 mM. The intestinal phase was conducted at pH 7.0 and 37 °C for 2 h under continuous agitation. All electrolyte compositions, enzyme activities, incubation times, pH conditions, and digestion procedures were established in accordance with the standardized INFOGEST 2.0 protocol to ensure methodological consistency and experimental reproducibility. At the end of each digestion stage, aliquots were collected, and enzymatic reactions were terminated by thermal inactivation at 90 °C for 10 min. The resulting digestates were freeze-dried and stored as stable powders until further physicochemical, antioxidant, antimicrobial, and cell-based analyses.

### 2.3. Fourier Transform Infrared Spectroscopy

Fourier Transform Infrared (FTIR) spectroscopy was used to evaluate the structural characteristics of collagen peptide samples using an Agilent Cary 630 spectrometer (Agilent Technologies, Santa Clara, CA, USA) equipped with a diamond ATR accessory. Analyses were performed on both undigested collagen peptide powders and samples obtained after in vitro gastrointestinal digestion. Prior to analysis, all samples were freeze-dried to ensure measurement consistency and minimize moisture-related spectral variations. FTIR spectra were recorded within a wavenumber range of 500–4000 cm^−1^ by directly scanning the dried samples without additional preparation. Attention was given to the characteristic amide regions, including amide A, amide I, amide II, and amide III bands, which are closely associated with collagen structural organization and protein secondary structure [16,17]. Variations in spectral profile and band intensity before and after gastrointestinal digestion were comparatively evaluated to investigate potential structural modifications in collagen peptide fractions induced by enzymatic digestion.

### 2.4. Molecular Weight Analysis

The molecular weight (MW) distribution of collagen peptide samples was determined by high-performance size exclusion chromatography (HP-SEC) according to the method described by Sheikha [18]., without modification. Analyses were performed using an Agilent 1200 HPLC system (Agilent Technologies, Waldbronn, Germany) equipped with a TSKgel G2000 SWXL size exclusion column (7.8 × 300 mm; Tosoh Bioscience, Tokyo, Japan). Prior to analysis, collagen peptide samples were dissolved in ultrapure water and filtered through a 0.45 μm membrane filter. A 20 μL aliquot of each sample was injected into the chromatographic system. Separation was carried out using an isocratic mobile phase consisting of KH_2_PO_4_, Na_2_HPO_4_, NaCl, benzoic acid, and ultrapure water at a flow rate of 0.5 mL/min. Peptides were detected at 214 nm using a UV detector (Agilent Technologies, Waldbronn, Germany). Molecular weight calibration was performed using porcine collagen peptide standards (GME) with known molecular weights of 1920, 2850, 3310, 4560, and 7765 Da. The average molecular weight of each sample was estimated from the calibration curve generated using the reference standards. In addition, peptide fractions were grouped into four molecular weight ranges (0–500, 500–1000, 1000–2000, and >2000 Da), and the relative proportion of each fraction was calculated from the chromatographic peak area (%) to evaluate changes in molecular weight distribution before and after simulated gastrointestinal digestion. All analyses were performed in triplicate.

### 2.5. Amino Acid Composition

The amino acid composition of collagen peptide samples was determined by high-performance liquid chromatography (HPLC). For the determination of most amino acids, samples were hydrolyzed with 6 N hydrochloric acid (HCl) at 110 °C for 16 h in sealed tubes to ensure complete protein hydrolysis. Following hydrolysis, the samples were diluted with deionized water and filtered to remove insoluble residues. Tryptophan, which is unstable under acidic hydrolysis conditions, was determined separately following alkaline hydrolysis. The resulting hydrolysates were analyzed using a High-Performance Liquid Chromatography (HPLC) system equipped with a Waters 410 fluorescence detector (Waters Corporation, Milford, MA, USA) and an AccQ-Tag column (3.9 × 150 mm). Amino acid separation was achieved using AccQ-Tag Eluent A and Eluent B, together with a secondary acidic eluent containing 60% acetonitrile at a flow rate of 1.0 mL/min [8].

### 2.6. Determination of Degree of Hydrolysis

The degree of hydrolysis (DH) of the collagen peptide samples was determined using the o-phthaldialdehyde (OPA) spectrophotometric method described by Nielsen et al. [19], with minor modifications. The OPA reagent was freshly prepared by dissolving sodium tetraborate decahydrate, sodium dodecyl sulfate (SDS), o-phthaldialdehyde (OPA), and dithiothreitol (DTT) in deionized water. A serine solution (0.1 g/L) was used as the calibration standard. For analysis, 3 mL of OPA reagent was mixed with 400 μL of deionized water (blank), serine standard, or sample solution. After incubation at room temperature for 2 min, absorbance was measured at 340 nm using a UV–visible spectrophotometer (V-650, Jasco Europe, Cremella, Italy). All measurements were performed in triplicate.

The degree of hydrolysis (%) was calculated using the equations below:

(1) $${\mathrm{Serine}\text{-}\mathrm{NH}}_{2}=\left[\frac{\left({\mathrm{OD}}_{sample}\;-\;{\mathrm{OD}}_{blank}\right)}{\left({\mathrm{OD}}_{standard}\;-\;{\mathrm{OD}}_{blank}\right)}\right]\times 0.9516\;\mathrm{meqv}/L\times \left[\frac{0.1\times 100}{X\times P}\right]$$

(2) $$h=\frac{\left({\mathrm{Serine}\text{-}\mathrm{NH}}_{2}\;-\;\beta \right)}{\alpha }\;$$

(3) $$DH\left(\%\right)=\frac{h}{h_{tot}}\times 100$$

Serine–NH_2_ represents the milliequivalents of free amino groups per gram of protein; *X* denotes the sample weight (g); *P* indicates the protein content (%); and 0.1 refers to the sample volume (L). *DH* was calculated using the *α*, *β*, and *h_tot_* constants proposed by Adler-Nissen [20] for gelatin-derived proteins (*α* = 0.796, *β* = 0.457, and *h_tot_* = 11.1 meq g^−1^ protein). Because all commercial collagen peptide products examined in this study were produced by enzymatic hydrolysis of bovine hide gelatin and, according to the manufacturers’ specifications, contained no added non-collagen ingredients, the same constants were used for all samples to ensure methodological consistency and direct comparability. Free amino acid correction was not applied, as all samples were analyzed under identical experimental conditions using the same analytical procedure.

### 2.7. Antioxidant Activity Assays (DPPH and ABTS^+^)

The antioxidant capacity of collagen peptide samples was evaluated using DPPH (2,2-diphenyl-1-picrylhydrazyl) and ABTS^+^ [2,2-azino-bis (3-ethylbenzothiazoline-6-sulfonic acid)] radical scavenging assays according to the method described by Chobdar Rahim et al. [21]. Trolox was used as the reference antioxidant standard for both assays. For the DPPH assay, a freshly prepared methanolic DPPH solution (6 × 10^−5^ M) was mixed with diluted sample solution at a ratio of 4.9 mL DPPH solution to 0.1 mL sample. The reaction mixtures were incubated in the dark at room temperature for 30 min, and absorbance was measured at 517 nm using a UV–visible spectrophotometer (Shimadzu UV-1240, Shimadzu Corporation, Kyoto, Japan). Antioxidant activity was expressed as Trolox equivalent antioxidant capacity (TEAC) in mg Trolox equivalents per 100 g sample (mg TE/100 g). For the ABTS^+^ assay, the radical cation was generated by reacting 7 mM ABTS^+^ with 2.45 mM potassium persulfate followed by incubation in the dark at room temperature for 12–16 h. Prior to analysis, the solution was diluted with distilled water to obtain an absorbance value of 0.700 ± 0.02 at 734 nm. Subsequently, 2 mL of the ABTS^+^ solution was mixed with 0.1 mL of sample solution and incubated in the dark for 6 min. Absorbance was recorded at 734 nm, and results were expressed as TEAC values in mM Trolox equivalents per 100 g sample.

In addition to TEAC values, DPPH radical scavenging activity was also expressed as percentage inhibition according to the following equation:

(4) $$\%\;\mathrm{Inhibition}=\frac{\left(A_{control}\;-\;A_{sample}\right)}{A_{control}}\times 100$$

where $A_{control}$ represents the absorbance of the radical solution without sample, and $A_{sample}$ corresponds to the absorbance of the reaction mixture containing both sample and radical solution.

### 2.8. Cell Viability Assay (MTT)

The cytocompatibility of commercial collagen peptide samples (RC, LC, HC, VC, and NC) and their corresponding simulated gastrointestinal digestates was evaluated using the MTT (3-(4,5-dimethylthiazol-2-yl)-2,5-diphenyltetrazolium bromide) assay on L929 fibroblast cells according to the method described by Mosmann [13]. Briefly, cells were seeded into 96-well culture plates at a density of 1 × 10^4^ cells/well and allowed to attach prior to treatment. Undigested and simulated gastrointestinal-digested collagen peptide samples prepared according to the INFOGEST 2.0 protocol were dissolved in complete DMEM medium and tested at final concentrations of 1, 3, and 5 mg/mL. These concentrations were selected to evaluate concentration-dependent cellular responses over a range commonly employed in previous in vitro studies of collagen peptides. The pH of the treatment media was adjusted and confirmed at 7.5 prior to cell exposure, whereas osmolarity was not measured separately. Following incubation periods of 1, 3, and 7 days, 100 μL of MTT solution (0.5 mg/mL) was added to each well and incubated for 4 h at 37 °C. The culture medium was subsequently removed, and the resulting formazan crystals were dissolved in 100 μL dimethyl sulfoxide (DMSO). To ensure complete solubilization, the plates were gently shaken for 30 min prior to measurement. Absorbance values were recorded at 570 nm using a microplate reader (Thermo Fisher Scientific, Vantaa, Finland). Cell viability was expressed as a percentage relative to untreated control cells, which were considered 100% viable, according to the following equation:

(5) $$\mathrm{Cell}\mathrm{Viability}\;(\%)=\left(\frac{A_{sample}}{A_{control}}\right)\times 100$$

$A_{sample}\;$represents the absorbance of treated cells and $A_{control}$ corresponds to the absorbance of untreated control cells.

### 2.9. Determination of Antimicrobial Activity

The antimicrobial activities of commercially available collagen peptide samples and their corresponding gastrointestinal digestates were evaluated using the agar disk diffusion method according to Bauer et al. [22]. Lyophilized samples were dissolved in sterile distilled water to obtain a final concentration of 100 mg/mL and sterilized by filtration through 0.22 μm membrane filters prior to analysis. Antimicrobial activity was tested against four pathogenic bacterial strains, including two Gram-positive bacteria, *Staphylococcus aureus* ATCC 25923 and *Enterococcus faecalis* ATCC 29212, and two Gram-negative bacteria, *Escherichia coli* ATCC 25922 and *Pseudomonas aeruginosa* ATCC 27853. Bacterial suspensions were prepared in sterile saline solution and adjusted to approximately 1–2 × 10^8^ CFU/mL, corresponding to the 0.5 McFarland standard. The assay was conducted using a modified Kirby–Bauer disk diffusion procedure. Briefly, bacterial cultures were uniformly spread onto Tryptic Soy Agar (TSA) plates using sterile swabs to obtain a homogenous bacterial lawn. Sterile cellulose disks (6 mm diameter) were loaded with 20 μL of sample solution and placed on the agar surface. Commercial antibiotic disks and sterile distilled water disks were used as positive and negative controls, respectively. To facilitate peptide diffusion within the agar matrix, plates were maintained at 4 °C for 2 h prior to incubation. Subsequently, the plates were incubated at 37 °C for 48 h, and inhibition zone diameters were measured in millimeters using a digital caliper (INSIZE Co., Ltd., Suzhou, China).

### 2.10. In Vitro Scratch (Wound Healing) Assay

The migratory behavior of L929 fibroblasts was evaluated using an in vitro scratch (wound healing) assay according to previously described methods [23,24]. Cells were cultured until reaching 90–100% confluence, after which a linear scratch was created using a sterile pipette tip. Detached cells and cellular debris were removed by washing with phosphate-buffered saline (PBS) [25]. To minimize the contribution of cell proliferation and ensure that wound closure predominantly reflected cell migration, all treatments were performed in low-serum culture medium containing 1% fetal bovine serum (FBS). Cells were treated with five commercial collagen peptide samples (RC, LC, HC, VC, and NC) in both undigested and simulated gastrointestinal-digested forms prepared according to the INFOGEST 2.0 protocol. Untreated cells served as the control group (*n* = 3). Microscopic images were captured at 0, 24, 48, and 72 h using an inverted microscope (Oxion Inverso, Euromex Microscopen B.V., Duiven, The Netherlands) for subsequent evaluation of wound closure and fibroblast migration. Wound closure percentage was calculated using the following equation:

(6) $$\mathrm{Wound}\mathrm{Closure}\;(\%)=\frac{\left(A_{0}-A_{t}\right)}{A_{0}}\times 100$$

where $A_{0}$ represents the initial wound area at 0 h and $A_{t}$ corresponds to the wound area measured at the respective time point.

### 2.11. Statistical Analysis

All experimental analyses were conducted using a completely randomized design consisting of three independent biological experiments, each performed with two technical replicates per treatment. Technical replicates were averaged prior to statistical analysis. Data were expressed as mean ± standard deviation. Statistical differences among sample groups were evaluated by one-way analysis of variance (ANOVA) followed by Duncan’s multiple range test for post hoc comparisons. Statistical analyses were performed using SPSS software (version 25.0; SPSS Inc., Chicago, IL, USA), and differences were considered statistically significant at *p* < 0.05.

## 3. Results

### 3.1. Chemical Composition of Commercial Collagen Peptide Samples

The chemical composition of commercially available collagen peptide samples is presented in Table 1. Crude protein content ranged from 88.58% to 94.37%, with no statistically significant differences among samples (*p* > 0.05), indicating a generally comparable protein enrichment across products. In contrast, moisture and ash contents differed significantly (*p* < 0.05), suggesting variability in processing conditions, drying efficiency, and mineral composition among commercial formulations. As expected for collagen-derived peptides, all samples exhibited relatively low lipid contents (0.38–0.57%).

Hydroxyproline content, one of the characteristic amino acids associated with collagen structure, also varied significantly among samples (*p* < 0.05). Among the evaluated samples, hydroxyproline content ranged from 7.63% to 9.07%, with HC exhibiting the highest value and NC the lowest. Hydroxyproline is a characteristic amino acid of collagen and is widely recognized as an indicator of collagen-derived proteins. Therefore, the observed differences may reflect variations in raw material characteristics and manufacturing processes, including the extent of hydrolysis. However, these differences should not be interpreted as direct evidence of overall product quality or biological performance, as multiple factors may influence the composition of commercial collagen peptide products.

Overall, although total protein contents were relatively similar among samples, differences in hydroxyproline, moisture, and ash levels demonstrate that commercially available collagen peptides are not compositionally uniform. Such compositional variability may contribute to differences in structural stability, digestion behavior, and functional properties observed in subsequent analyses. The compositional findings obtained in this study are consistent with previous reports on collagen peptides and commercially available collagen peptide products [2,3,26,27], supporting the reliability of the analytical results.

### 3.2. FTIR Analysis

**Figure 1:**
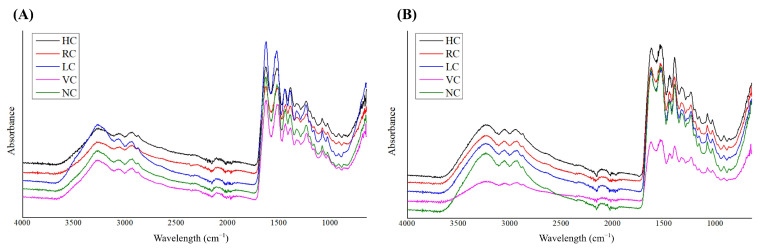
FTIR spectra of collagen peptides: (**A**) pre-digestion samples; (**B**) post-digestion samples.

The FTIR spectra of collagen peptide samples before and after simulated gastrointestinal digestion are presented in Figure 1. All samples exhibited the characteristic collagen-associated amide bands (amide A, amide I, amide II, and amide III), confirming the typical spectral features of collagen-derived peptides [16].

Following simulated gastrointestinal digestion, all samples showed changes in band intensity and moderate peak broadening, particularly in the amide I and amide II regions. These spectracharacteristicsith enzymatic hydrolysis and modifications in the chemical environment of peptide bonds during digestion [17,28]. Although the characteristic amide bands remained detectable after digestion, FTIR analysis alone does not allow definitive conclusions regarding peptide structural preservation or biological functionality. Therefore, the observed spectral differences should be interpreted as evidence of digestion-induced molecular changes rather than direct indicators of peptide stability or bioactive properties.

### 3.3. Evaluation of Degree of Hydrolysis and Molecular Weight

The combined evaluation of degree of hydrolysis (DH) and molecular weight (MW) of collagen peptide samples before and after simulated gastrointestinal digestion is presented in Table 2. Distinct differences were observed among the commercial samples in both DH and MW values, indicating considerable variability in peptide structure and hydrolysis profiles.

Initially, HC exhibited the highest DH value (13.40%) together with the lowest MW (1712 Da), suggesting the presence of relatively smaller peptide fractions generated through a more controlled hydrolysis process. In contrast, Vital Collagen (VC) showed the lowest initial DH (4.30%) and the highest MW (3576 Da), indicating the predominance of larger and less hydrolyzed peptide structures. Similar relationships between hydrolysis degree and peptide size distribution have previously been reported for collagen-derived peptides, where lower MW fractions were associated with more advanced enzymatic hydrolysis and improved peptide accessibility [4,10]. Following simulated gastrointestinal digestion, all samples demonstrated increased DH values accompanied by reductions in MW, confirming further enzymatic peptide breakdown during digestion. However, the magnitude of these structural changes varied markedly among samples. VC exhibited a pronounced increase in DH (61.62%) together with a substantial reduction in MW, suggesting more extensive hydrolysis and the formation of smaller peptide fractions following simulated gastrointestinal digestion. Such extensive degradation may indicate that larger peptide fractions present prior to digestion underwent rapid hydrolysis under gastrointestinal conditions. In contrast, HC showed a comparatively moderate increase in DH together with a more limited reduction in MW, indicating relatively controlled digestion behavior and improved structural stability during enzymatic hydrolysis. The smaller structural changes observed in HC may suggest that its peptide fractions had already undergone a more advanced and balanced hydrolysis prior to gastrointestinal digestion, thereby reducing susceptibility to excessive enzymatic degradation. Previous studies have similarly demonstrated that the initial molecular organization and hydrolysis profile of collagen peptides strongly influence digestion behavior, peptide stability, and structural resistance during gastrointestinal exposure [11].

Overall, the findings suggest a relationship between the initial peptide characteristics and their response to simulated gastrointestinal digestion. Samples characterized by higher initial MW and lower DH values generally exhibited greater changes in MW and DH during digestion, whereas samples with relatively lower initial MW and higher DH values showed comparatively smaller changes under the applied digestion conditions. Similar observations have been reported in previous studies, indicating that hydrolysis conditions influence peptide size distribution, digestion behavior, and the in vitro functional properties of collagen-derived peptides [2,4]. Collectively, these findings suggest that a balanced hydrolysis profile, rather than extensive hydrolysis alone, may contribute to differences in the in vitro functional properties of collagen peptide products following simulated gastrointestinal digestion.

**Figure 2:**
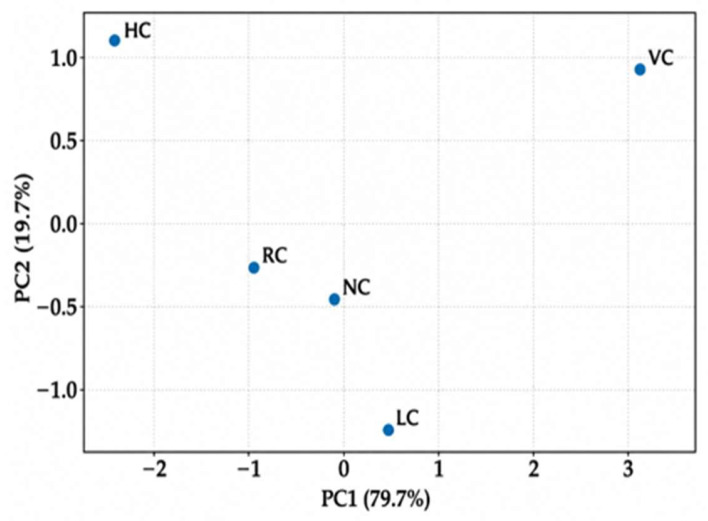
PCA molecular weight and hydrolysis parameters.

Principal component analysis (PCA) further supported the observed differences among collagen peptide samples based on their hydrolysis and molecular weight characteristics (Figure 2). VC formed a distinct cluster associated with high molecular weight and extensive digestion-induced hydrolysis, indicating greater susceptibility to enzymatic degradation. In contrast, HC was positioned separately from the other commercial samples, reflecting a comparatively controlled hydrolysis profile and improved structural stability during gastrointestinal digestion. Meanwhile, Reborn Collagen (RC), Lapi Collagen (LC), and NC exhibited relatively closer clustering patterns, suggesting more similar structural and digestion behaviors.

The molecular weight distribution profiles demonstrated that all collagen peptide samples underwent progressive fragmentation during simulated gastrointestinal digestion, resulting in increased proportions of low-molecular-weight peptides, particularly within the 0–500 Da and 500–1000 Da ranges Table 3. Among the evaluated samples, VC exhibited the most pronounced fragmentation pattern, characterized by a substantial reduction in peptides above 2000 Da and a marked increase in lower-molecular-weight fractions after digestion. LC and RC also showed notable shifts toward intermediate and lower molecular weight peptides, whereas NC maintained relatively higher proportions of larger peptide fractions even after digestion. In comparison, HC exhibited a more balanced molecular weight profile both before and after digestion, characterized by a controlled increase in small peptide fractions while still preserving a considerable proportion of intermediate-sized peptides (1000–2000 Da). This relatively stable peptide distribution may indicate improved structural stability and more controlled hydrolysis behavior under gastrointestinal conditions.

### 3.4. Amino Acid Profile of Commercial Collagen Peptide Samples

The amino acid composition of collagen peptide samples before and after simulated gastrointestinal digestion is presented in Table 4. Overall, gastrointestinal digestion did not result in substantial losses in total amino acid content, and the general amino acid profiles remained largely preserved across all samples. These findings suggest that the nutritional characteristics of collagen peptides were generally maintained under simulated gastrointestinal conditions.

Among the detected amino acids, glycine, proline, and hydroxyproline were the predominant components in all samples, which is consistent with the characteristic amino acid composition of collagen-derived peptides. These amino acids play a critical role in maintaining collagen structure through the repetitive Gly–X–Y sequence motif, where X and Y are commonly occupied by proline and hydroxyproline residues [26]. Glycine contributes to the compact arrangement of the collagen helix, whereas proline and hydroxyproline are associated with structural rigidity and stabilization of the triple-helical organization. Despite the overall similarity in amino acid composition among samples, differences were observed in compositional stability following gastrointestinal digestion. HC exhibited a comparatively stable distribution of both total and essential amino acids before and after digestion, indicating a relatively preserved peptide organization during enzymatic exposure. In contrast, several other samples demonstrated more pronounced fluctuations in specific amino acid levels, suggesting differences in digestion susceptibility and peptide fragmentation behavior. Such variations may be associated with differences in initial peptide size distribution, hydrolysis conditions, and structural organization among commercial collagen peptide products. Notably, the preservation of glycine, proline, and hydroxyproline after digestion further supports the structural resilience of collagen-derived peptides under gastrointestinal conditions. Similar findings have previously been reported in protein hydrolysates and collagen peptides, where simulated digestion resulted in limited alterations in overall amino acid composition despite structural modifications at the peptide level [4,29].

Overall, the findings indicate that gastrointestinal digestion does not substantially alter the total amino acid composition of collagen peptides; however, it may influence the relative distribution of specific amino acids depending on the initial characteristics of the samples. These results further emphasize the importance of controlled hydrolysis conditions in maintaining the amino acid profile and may contribute to differences in the in vitro functional properties of collagen peptides.

### 3.5. Evaluation of Antioxidant Activity Before and After Digestion

The antioxidant capacities of the commercial collagen peptide samples before and after simulated gastrointestinal digestion are presented in Table 5. Significant differences (*p* < 0.05) were observed among the samples in both DPPH and ABTS radical scavenging assays, indicating that simulated gastrointestinal digestion affected the in vitro radical scavenging capacity of the collagen peptide samples to different extents. Prior to digestion, HC exhibited the highest antioxidant activity in both assays and retained comparatively higher antioxidant values following gastrointestinal digestion. Similar digestion-dependent differences in the antioxidant capacity of collagen-derived peptides have previously been reported and have been associated with variations in peptide composition and molecular characteristics generated during enzymatic hydrolysis [4,30].

In contrast, LC exhibited a different antioxidant response. Although its initial antioxidant activity was relatively low, a marked increase was observed following simulated gastrointestinal digestion. This increase may be associated with the generation of smaller peptide fragments during enzymatic hydrolysis, which could contribute to enhanced radical scavenging capacity under the assay conditions. Similar digestion-dependent increases in the antioxidant capacity of collagen peptides have been reported previously and have been attributed to changes in peptide size and amino acid composition resulting from enzymatic digestion [31].

In contrast, RC and VC exhibited lower antioxidant values following simulated gastrointestinal digestion. In particular, VC showed a marked increase in the degree of hydrolysis (DH) together with a reduction in average molecular weight (MW); however, this was not accompanied by a proportional increase in DPPH or ABTS radical scavenging capacity. This observation suggests that changes in DH and MW alone are not sufficient to explain the antioxidant response of collagen peptide samples, and that additional factors, such as peptide composition, may also contribute to the observed differences. Similar findings have been reported in collagen peptides, where the relationship between hydrolysis, molecular weight distribution, and antioxidant capacity was not always linear [30,32]. In contrast, NC exhibited an increase in antioxidant activity after digestion despite its relatively low initial values. This result indicates that simulated gastrointestinal digestion influenced the in vitro radical scavenging capacity of the sample, consistent with previous observations reported for collagen peptides following enzymatic digestion [31].

**Figure 3:**
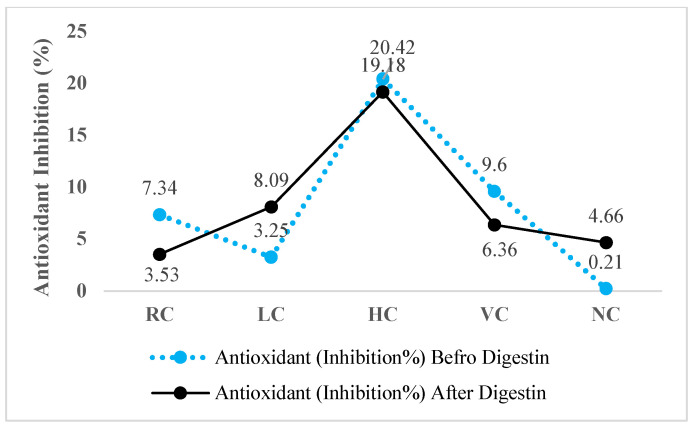
Changes in antioxidant inhibition (%) of commercial collagen peptide samples before and after simulated gastrointestinal digestion.

Overall, the results demonstrate that simulated gastrointestinal digestion influenced the in vitro radical scavenging capacity of the commercial collagen peptide samples, although the magnitude and direction of these changes differed among products. These differences may be associated with variations in molecular weight distribution and the degree of hydrolysis observed after digestion. As shown in Figure 3, the antioxidant responses varied among the evaluated samples, indicating that gastrointestinal digestion affected their chemical antioxidant properties to different extents. However, since DPPH and ABTS are in vitro chemical assays, these findings should be interpreted as comparative radical scavenging responses rather than direct evidence of biological efficacy or in vivo antioxidant activity.

The relationship between the degree of hydrolysis (DH), molecular weight (MW), and antioxidant activity was not strictly linear [33]. In some samples, increases in DH accompanied by reductions in MW were associated with higher in vitro radical scavenging capacity, whereas this trend was not consistently observed across all products. For example, VC exhibited a marked increase in DH together with a reduction in MW, but no proportional increase in DPPH or ABTS values was observed. These findings suggest that changes in DH and MW alone do not fully explain the antioxidant responses of collagen peptide samples, and that additional factors related to peptide composition may also contribute. Therefore, the antioxidant capacity of collagen peptide products appears to depend on multiple characteristics generated during enzymatic hydrolysis rather than on the extent of hydrolysis alone.

### 3.6. Antimicrobial Activity

**Figure 4:**
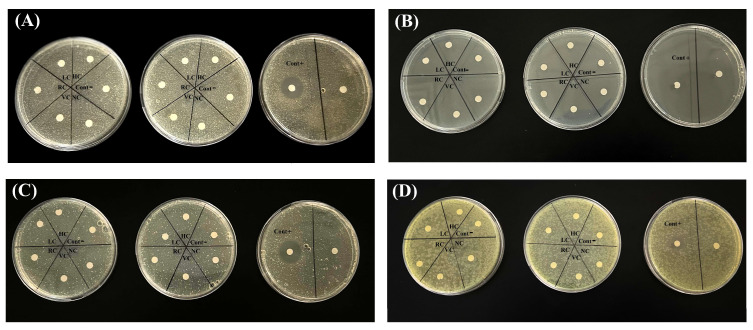
Evaluation of the antimicrobial activity of undigested and digested commercial collagen peptides via the disk diffusion assay after 48 h of incubation. (**A**) *Staphylococcus aureus*; (**B**) *Enterococcus faecalis*; (**C**) *Escherichia coli*; and (**D**) *Pseudomonas aeruginosa*. The panels show the inhibition zones (ZOI) obtained for the undigested commercial collagen peptide samples (RC, LC, HC, VC, and NC), their corresponding samples after simulated gastrointestinal digestion, and the positive and negative controls.

The antimicrobial activities of the commercial collagen peptide samples and their corresponding gastrointestinal digestates were evaluated against selected Gram-positive and Gram-negative bacteria using the agar disk diffusion assay. No detectable inhibition zones were observed for any of the collagen peptide samples before or after simulated gastrointestinal digestion (Figure 4). The positive controls produced clear inhibition zones against all tested microorganisms, confirming the validity of the assay.

The absence of antimicrobial activity indicates that the evaluated collagen peptide samples did not exhibit measurable antibacterial effects under the tested conditions. This finding is consistent with previous reports showing that antimicrobial activity of collagen-derived peptides depends on specific peptide characteristics, hydrolysis conditions, and the presence of particular peptide sequences rather than on hydrolysis alone [4,34,35,36]. Therefore, under the experimental conditions employed in this study, the commercial collagen peptide products demonstrated no detectable antimicrobial activity.

### 3.7. Cytocompatibility and Dose-Dependent Cell Viability (MTT Assay)

**Figure 5:**
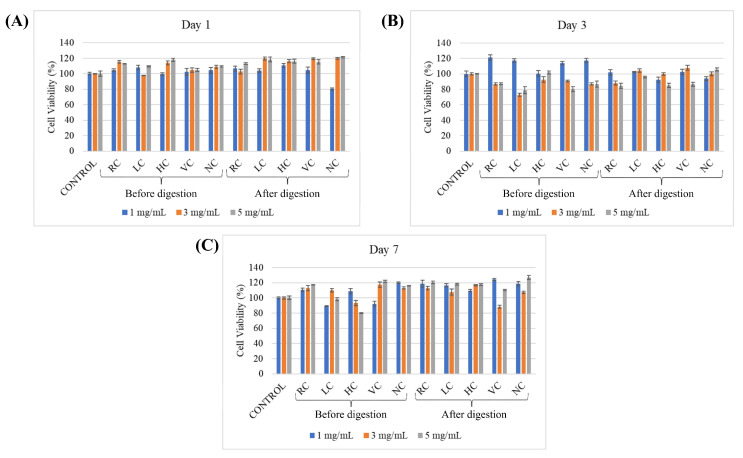
The effects of undigested (before digestion) and digested (after digestion) commercial collagen peptides (RC, LC, HC, VC, NC) on L929 fibroblast cell viability at concentrations of 1, 3, and 5 mg/mL. (**A**) Cell viability percentages on Day 1; (**B**) cell viability percentages on Day 3; (**C**) cell viability percentages on Day 7. Data are presented as the mean percentage of viability relative to the untreated control ± SD.

The in vitro cytocompatibility of five commercially available collagen peptides (RC, LC, HC, VC, and NC) and their corresponding gastrointestinal digestates were evaluated using L929 fibroblast cells over a 7-day incubation period. Across all tested concentrations and incubation times, cell viability remained above the 70% threshold defined by ISO 10993-5 standards [37], indicating that none of the tested samples exhibited cytotoxic effects under the experimental conditions. As presented in Figure 5, all collagen peptide samples demonstrated high initial cellular compatibility on Day 1, with viability values generally ranging between 95% and 120% for both undigested and digested forms. However, by Day 3, concentration-dependent differences in metabolic activity became more apparent. Samples applied at lower concentrations generally maintained elevated cellular activity, whereas higher concentrations produced a temporary reduction in viability in several groups. Nevertheless, viability values remained within the non-cytotoxic range throughout the experimental period.

The observed temporal behavior characterized by an initially stable phase followed by a moderate reduction and subsequent recovery in viability may indicate a cellular adaptation response to peptide-rich environments. Exposure to relatively high concentrations of complex protein peptides may transiently influence osmotic balance, amino acid transport, or cellular metabolic regulation, thereby temporarily affecting mitochondrial activity and proliferation behavior. Importantly, the MTT assay primarily reflects mitochondrial reductase activity rather than direct cell death [13]. Therefore, the temporary reductions observed on Day 3 likely represent short-term metabolic adaptation rather than irreversible cytotoxic damage. As incubation progressed, most samples demonstrated recovery or enhancement of cellular activity by Day 7, indicating that fibroblasts adapted successfully to the collagen peptide environment.

Among the tested samples, gastrointestinal digestion appeared to positively influence cellular responses in several groups. For example, the digested HC sample exhibited improved viability compared with its undigested counterpart at higher concentrations, suggesting that enzymatic digestion may enhance peptide bioavailability and cellular interaction. Simulated gastrointestinal hydrolysis is known to reduce peptide molecular size and generate more accessible bioactive peptide fractions, thereby potentially improving cellular uptake and biological activity [15,38]. Differences in proliferation dynamics among samples may additionally reflect variations in peptide composition, hydrolysis profiles, amino acid distribution, and raw material characteristics. Notably, digested VC and NC samples exhibited relatively elevated proliferative responses at later incubation stages, whereas HC demonstrated a comparatively more stable and controlled response profile throughout the experimental period. Such differences suggest that gastrointestinal digestion may modulate the biological activity of collagen-derived peptides in a sample-dependent manner.

Overall, the findings demonstrate that all evaluated collagen peptides exhibit good cytocompatibility and are potentially suitable for nutraceutical and biomedical applications. Furthermore, gastrointestinal digestion appears to enhance the biological accessibility and cellular interaction potential of collagen-derived peptide fractions, thereby contributing positively to their functional bioactivity.

### 3.8. Fibroblast Migration and Wound Healing Capacity

**Figure 6:**
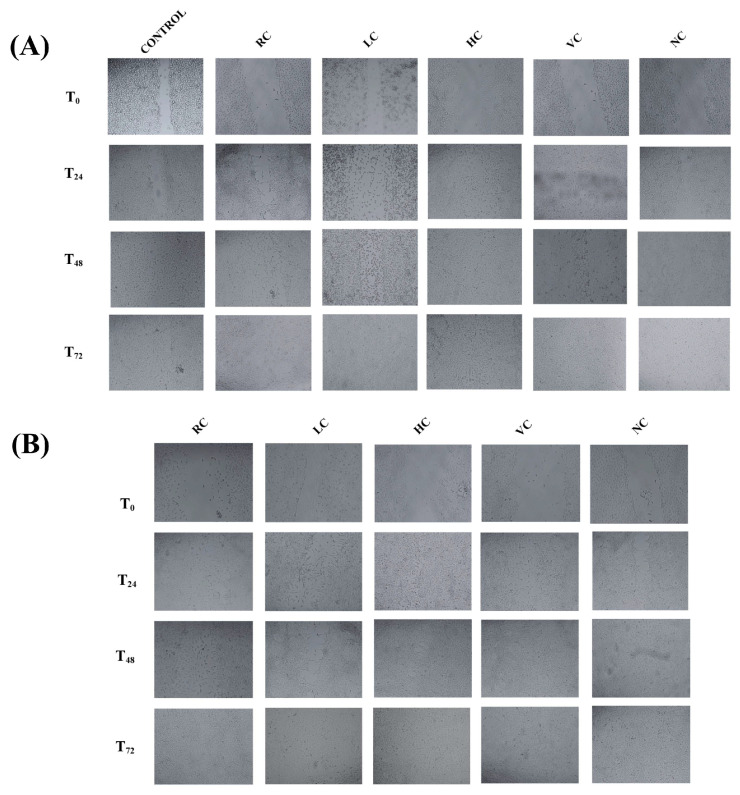
Representative phase-contrast micrographs of the in vitro scratch (wound healing) assay demonstrating L929 fibroblast migration. (**A**) Fibroblast migration over 72 h in response to undigested collagen peptides. (**B**) Migratory responses following treatment with simulated gastrointestinal digestates of the corresponding samples. Images were captured at 0, 24, 48, and 72 h post-scratch using an inverted microscope. The untreated control group represents basal migration under low-serum conditions.

The impact of collagen peptides on cell motility and time-dependent migration kinetics was evaluated by quantitatively analyzing cellular advancement within the scratched areas, and percentage wound closure rates were calculated relative to the initial scratch area (T0). Under reduced serum conditions (1% FBS), commonly used to minimize cell proliferation during scratch assays, wound closure is considered to predominantly reflect cellular migration, although a contribution of cell proliferation cannot be completely excluded (Figure 6).

**Figure 7:**
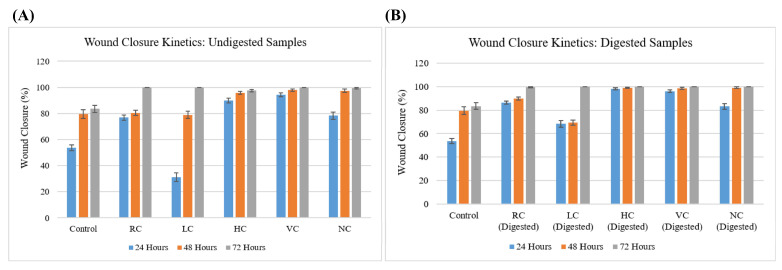
Quantitative analysis of wound closure kinetics in L929 fibroblasts treated with collagen peptides. (**A**) Percentage wound closure of undigested commercial collagen peptides (RC, LC, HC, VC, NC). (**B**) Wound closure kinetics following treatment with simulated gastrointestinal digestates (INFOGEST protocol) of the respective samples. Data are expressed as mean wound closure (%) ± SD (*n* = 3).

The control group exhibited gradual wound closure rates of 53.79% ± 2.14, 79.49% ± 3.25, and 83.45% ± 2.80 at 24, 48, and 72 h, respectively, confirming that wound closure under low-serum conditions was primarily associated with directed fibroblast migration rather than excessive proliferation [24]. The results obtained demonstrated that collagen source and peptide structure exerted sample-dependent effects on fibroblast motility (Figure 7A). Among the undigested peptides, VC and HC exhibited the most rapid early-stage migration responses, reaching closure rates of 94.42% ± 1.45 and 89.80% ± 1.85 within the first 24 h, respectively. Microscopic observations further revealed that fibroblasts in these groups maintained a more uniform migration front together with their characteristic spindle-shaped morphology during wound closure.

The undigested NC sample also demonstrated high closure activity, reaching 97.37% ± 1.34 at 48 h. However, microscopic evaluation indicated increased local cell clustering and less organized cellular alignment during migration. This finding may suggest that excessively rapid closure does not always reflect coordinated wound healing behavior and may instead be associated with irregular cellular organization [39].

Conversely, the undigested LC group exhibited the slowest initial migration profile, reaching only 31.11% ± 3.42 wound closure within the first 24 h, which remained below the basal control group. This delayed response may be associated with the limited accessibility of specific peptide motifs involved in fibroblast adhesion and migration processes [40]. However, with prolonged incubation up to 72 h, all undigested hydrolysate groups, including RC and LC, achieved wound closure rates between 97% and 100%, exceeding the control group (83.45% ± 2.80). These findings suggest that collagen-derived peptides may contribute to long-term fibroblast migration through integrin-mediated signaling pathways associated with extracellular matrix interactions [14,40].

Simulated gastrointestinal digestion performed according to the INFOGEST protocol notably enhanced fibroblast migration and wound closure kinetics in all treatment groups (Figure 7B). The most pronounced improvement was observed in the LC group, where wound closure increased from 31.11% in the undigested form to 68.35% ± 2.85 following digestion at the same evaluation time point. Similarly, the digested RC group demonstrated accelerated migration kinetics, reaching 86.45% ± 1.50 closure at 24 h. Among the digested samples, HC and VC exhibited the highest early-stage migratory responses, achieving near-complete wound closure at 24 h with rates of 98.06% ± 0.85 and 96.21% ± 1.15, respectively. Fibroblasts treated with digested HC maintained a more homogeneous migration pattern together with preserved spindle-shaped morphology throughout the healing process. The digested NC group also maintained high migratory activity, reaching 99.23% ± 0.60 closure at 48 h. At the molecular level, gastrointestinal enzymes such as pepsin and pancreatin hydrolyze collagen macromolecules into smaller peptide fractions. Previous studies have suggested that certain low-molecular-weight collagen-derived peptides, including di- and tripeptides such as Pro-Hyp, may contribute to fibroblast chemotaxis and migration [41,42]. However, peptide identification or sequencing was not performed in the present study; therefore, the involvement of specific peptide sequences remains hypothetical and requires further investigation.

Overall, the results demonstrate that the wound healing potential of commercial collagen peptides is influenced by both peptide composition and gastrointestinal digestion behavior. While all digested samples exhibited improved migratory activity compared to their undigested forms, digested HC displayed a more balanced and structurally stable migration profile, suggesting its potential suitability for tissue regeneration and biomaterial-related applications.

## 4. Conclusions

This study provides a comparative in vitro evaluation of commercially available collagen peptide products by integrating compositional characterization, molecular weight distribution, structural analysis, antioxidant activity, and cell-based assays before and after simulated gastrointestinal digestion. Although all evaluated products exhibited acceptable compositional quality and favorable cellular responses, differences were observed in their structural characteristics and in vitro biological responses following digestion. The findings suggest that simulated gastrointestinal digestion influences the physicochemical and biological properties of collagen peptide products to different extents. Variations in molecular weight distribution, degree of hydrolysis, and structural characteristics may contribute to the observed differences in in vitro antioxidant activity and cellular responses among the evaluated products. However, these associations were not consistent across all samples, highlighting the complexity of collagen peptide functionality and the importance of product-specific evaluation rather than generalized assumptions.

Overall, this study provides a comparative dataset that contributes to the characterization of commercially available collagen peptide products and demonstrates the value of integrated analytical approaches for their comparative in vitro evaluation. Nevertheless, the biological relevance of the present findings requires confirmation through peptide characterization, bioavailability studies, animal models, and well-designed clinical investigations before conclusions regarding physiological efficacy or potential health benefits can be drawn.

## 5. Limitations

This study has several limitations that should be considered when interpreting the findings. First, the evaluated collagen peptide products were commercially available products obtained from the Turkish market, and each product was represented by a single production batch; therefore, batch-to-batch variability was not assessed. Second, although all products consisted of bovine collagen peptides without added functional ingredients, differences in manufacturing processes may have contributed to the observed variability among products. Third, peptide sequence identification was not performed; therefore, the specific peptides responsible for the observed biological responses could not be identified. Finally, the present study was limited to in vitro analyses under simulated gastrointestinal conditions. Consequently, peptide absorption, bioavailability, and physiological efficacy require confirmation through animal studies and well-designed clinical investigations. In addition, antimicrobial activity was evaluated using the agar disk diffusion assay, which has inherent limitations for peptide-based compounds due to restricted diffusion in agar. Future studies employing broth microdilution or minimum inhibitory concentration (MIC) assays may provide a more comprehensive evaluation.

## Figures and Tables

**Table 1:** Chemical composition of collagen peptide samples.

Sample	Crude Protein (%)	Moisture (%)	Ash (%)	Fat (%)	Hydroxyproline (%)
RC	90.12 ± 3.54 ^a^ *	4.04 ± 0.13 ^d^	0.94 ± 0.04 ^bc^	0.57 ± 0.07 ^c^	8.25 ± 0.14 ^b^
LC	91.38 ± 2.38 ^a^	3.67 ± 0.06 ^c^	0.86 ± 0.03 ^b^	0.42 ± 0.06 ^ab^	8.15 ± 0.14 ^b^
HC	94.37 ± 3.12 ^a^	3.18 ± 0.07 ^a^	0.72 ± 0.04 ^a^	0.38 ± 0.04 ^a^	9.07 ± 0.14 ^c^
VC	91.49 ± 4.04 ^a^	3.41 ± 0.09 ^b^	0.85 ± 0.03 ^b^	0.44 ± 0.04 ^ab^	8.38 ± 0.34 ^b^
NC	88.58 ± 2.64 ^a^	4.73 ± 0.06 ^e^	1.01 ± 0.07 ^c^	0.50 ± 0.03 ^bc^	7.63 ± 0.11 ^a^

* Indicates a statistically significant difference compared with the control group (*p* < 0.05). Different letters within each column indicate statistically significant differences among the commercial collagen peptide samples (*p* < 0.05)).

**Table 2:** Changes in DH and MW of collagen peptides during gastrointestinal digestion.

Sample	Initial DH (%)	MW	Post-Digestion DH (%)	Post-Digestion MW
RC	9.41 ± 0.05	1874	15.93 ± 0.69	1507
LC	5.70 ± 0.07	2188	15.16 ± 0.24	1574
HC	13.40 ± 0.18	1712	19.38 ± 0.53	1413
VC	4.30 ± 0.07	3576	61.62 ± 1.96	1578
NC	8.21 ± 0.13	2326	16.67 ± 0.57	1536

**Table 3:** Molecular weight distribution (%) of commercial collagen peptide samples before and after simulated gastrointestinal digestion.

Molecular Weight Range (Da)	Before Digestion					After Digestion				
	RC	LC	HC	VC	NC	RC	LC	HC	VC	NC
0–500	3.3	4.1	5.5	1.7	2.5	6.4	5.3	8.0	5.4	5.6
500–1000	9.3	10.3	15.8	4.9	7.7	15.9	19.2	23.5	13.7	14.3
1000–2000	35.4	28.4	45.1	16.3	33.2	45.5	47.6	42.0	28.3	35.4
>2000	52.0	57.2	33.6	77.1	56.6	32.2	27.9	26.5	52.6	44.7

**Table 4:** Amino acid composition of collagen peptides (g/100 g protein).

Amino Acid	Initial Amino Acid Composition					Post-Digestion Amino Acid Composition				
	RC	LC	HC	VC	NC	RC	LC	HC	VC	NC
Aspartic Acid	5.753	5.623	6.083	5.845	6.018	5.624	5.284	5.106	5.635	7.108
Glutamic Acid	10.642	9.711	10.153	8.832	8.183	9.4607	10.488	9.287	9.610	9.723
Serine	4.386	3.005	2.629	3.435	2.552	3.475	3.000	1.008	3.624	3.457
Histidine *	1.118	1.321	1.061	1.003	1.056	1.042	1.004	1.019	1.103	1.001
Glycine	20.102	21.816	22.676	20.988	20.38	22.852	22.482	23.445	20.478	19.639
Threonine *	1.135	1.002	1.098	1.548	1.454	1.996	2.000	1.814	1.036	2.027
Citrulline	0.406	0.509	0.508	0.524	2.420	0.613	0.563	0.694	0.694	0.720
Arginine	7.816	8.461	7.743	7.184	7.527	8.522	8.832	7.082	8.919	7.911
Alanine	9.701	9.139	9.003	8.641	9.234	7.671	7.767	5.634	5.976	8.379
Tyrosine	0.600	0.512	0.520	0.526	0.521	0.519	0.503	0.359	1.619	2.824
Cystine	<0.029	<0.030	<0.030	<0.030	<0.030	<0.030	<0.030	<0.030	<0.030	<0.030
Valine *	2.136	1.528	1.688	1.544	2.462	1.700	2.127	2.600	1.490	1.754
Methionine *	0.652	0.593	0.402	0.806	0.664	0.661	0.627	0.888	0.419	1.958
Tryptophan *	0.732	0.738	0.849	0.819	0.921	0.801	0.813	0.827	0.942	0.900
Phenylalanine *	2.516	3.887	2.311	2.009	1.663	3.332	3.000	2.235	2.591	2.831
Isoleucine *	1.718	1.839	2.095	1.515	1.427	1.541	1.384	1.021	1.645	1.409
Ornithine	<0.029	<0.029	<0.029	<0.029	<0.029	<0.029	<0.029	<0.029	<0.029	<0.029
Leucine *	3.142	2.801	3.593	3.745	3.630	2.397	2.457	2.743	2.534	2.524
Lysine *	4.106	4.118	4.299	4.017	4.258	4.263	4.018	4.258	6.368	4.983
Hydroxyproline	7.731	8.873	9.164	8.128	7.916	10.000	8.976	10.008	9.187	5.187
Sarcosine	<0.009	<0.009	<0.009	<0.009	<0.009	<0.009	<0.009	<0.009	<0.009	<0.009
Proline	12.918	12.984	13.943	12.845	11.972	12.741	12.412	13.129	12.001	11.211
Total essential amino acid **	17.255	17.827	17.396	17.006	17.530	17.733	17.430	17.405	17.738	17.347
Total amino acids *	99.47	99.32	99.88	99.61	99.32	99.23	99.80	99.81	99.90	99.70

* Essential amino acids. ** Total essential amino acid.

**Table 5:** Antioxidant capacities of collagen peptides.

Sample	Antioxidant Activity			
	Before Digestion		After Digestion	
	(DPPH) (mg Trolox/100 g Sample)	(ABTS^+^) (mg Trolox/100 g Sample)	(DPPH) (mg Trolox/100 g Sample)	(ABTS^+^) (mg Trolox/100 g Sample)
RC	24.65 ± 2.31 ^c^	21.78 ± 1.02 ^c^	11.85 ± 0.65 ^a^	10.51 ± 0.77 ^a^
LC	10.91 ± 1.36 ^b^	9.51 ± 0.97 ^b^	27.17 ± 0.95 ^d^	25.53 ± 1.04 ^d^
HC	67.97 ± 4.24 ^e^	60.32 ± 3.84 ^e^	64.41 ± 1.54 ^e^	55.82 ± 3.16 ^e^
VC	32.24 ± 2.62 ^d^	28.53 ± 2.27 ^d^	21.36 ± 1.78 ^c^	18.77 ± 1.94 ^c^
NC	0.10 ± 0.02 ^a^	0.50 ± 0.00 ^a^	15.65 ± 0.67 ^b^	13.77 ± 0.90 ^b^

Different letters within each column indicate statistically significant differences (*p* < 0.05).

## Data Availability

The data presented in this study are available on reasonable request from the corresponding author. The data are not publicly available due to confidentiality restrictions related to commercially available products and industrial research.

## Abbreviations

The following abbreviations are used in this manuscript:

AOAC	Association of Official Analytical Chemists
DPPH	2,2-diphenyl-1-picrylhydrazyl
ABTS	2,2-azino-bis(3-ethylbenzothiazoline-6-sulfonic acid)
OPA	o-phthaldialdehyde
SDS	Sodium dodecyl sulfate
DTT	Dithiothreitol
HCl	Hydrochloric acid
SSF	Simulated salivary fluid
SGF	Simulated gastric fluid
SIF	Simulated intestinal fluid
TEAC	Trolox equivalent antioxidant capacity
MTT	3-(4,5-dimethylthiazol-2-yl)-2,5-diphenyltetrazolium bromide
DMSO	dimethyl sulfoxide
TSA	Tryptic Soy Agar
PBS	Phosphate-buffered saline
FBS	Fetal bovine serum
DH	Degree of hydrolysis
LC	Reborn Collagen
HC	Halavet Collagen
LC	Lapi Collagen
VC	Vital Collagen
NC	Nature’s Supreme Collagen
FTIR	Fourier transform infrared spectroscopy
HPLC	High-performance liquid chromatography
MW	Molecular weight
MN	Molecular weight distribution
ANOVA	Analysis of variance
PCA	Principal component analysis
SD	Standard Deviation
